# Blunted Neuronal Calcium Response to Hypoxia in Naked Mole-Rat Hippocampus

**DOI:** 10.1371/journal.pone.0031568

**Published:** 2012-02-21

**Authors:** Bethany L. Peterson, John Larson, Rochelle Buffenstein, Thomas J. Park, Christopher P. Fall

**Affiliations:** 1 Department of Biological Sciences, University of Illinois at Chicago, Chicago, Illinois, United States of America; 2 Laboratory of Integrative Neuroscience, University of Illinois at Chicago, Chicago, Illinois, United States of America; 3 Psychiatric Institute, Department of Psychiatry, University of Illinois at Chicago, Chicago, Illinois, United States of America; 4 Department of BioEngineering, University of Illinois at Chicago, Chicago, Illinois, United States of America; 5 Barshop Institute and Department of Physiology, University of Texas Health Science Center at San Antonio, San Antonio, Texas, United States of America; 6 Department of Computer Science, Georgetown University, Washington, D. C., United States of America; Seattle Children's Research Institute, United States of America

## Abstract

Naked mole-rats are highly social and strictly subterranean rodents that live in large communal colonies in sealed and chronically oxygen-depleted burrows. Brain slices from naked mole-rats show extreme tolerance to hypoxia compared to slices from other mammals, as indicated by maintenance of synaptic transmission under more hypoxic conditions and three fold longer latency to anoxic depolarization. A key factor in determining whether or not the cellular response to hypoxia is reversible or leads to cell death may be the elevation of intracellular calcium concentration. In the present study, we used fluorescent imaging techniques to measure relative intracellular calcium changes in CA1 pyramidal cells of hippocampal slices during hypoxia. We found that calcium accumulation during hypoxia was significantly and substantially attenuated in slices from naked mole-rats compared to slices from laboratory mice. This was the case for both neonatal (postnatal day 6) and older (postnatal day 20) age groups. Furthermore, while both species demonstrated more calcium accumulation at older ages, the older naked mole-rats showed a smaller calcium accumulation response than even the younger mice. A blunted intracellular calcium response to hypoxia may contribute to the extreme hypoxia tolerance of naked mole-rat neurons. The results are discussed in terms of a general hypothesis that a very prolonged or arrested developmental process may allow adult naked mole-rat brain to retain the hypoxia tolerance normally only seen in neonatal mammals.

## Introduction

Naked mole-rats (*Heterocephalus glaber*) initially received a great deal of attention when scientists discovered that they had a eusocial lifestyle similar to that of bees and termites [1]. Since then, a number of additional remarkable characteristics have been identified in this species [2]–[8]. Naked mole-rats are the only known poikilothermic mammals [9], and they live an extraordinarily long life (∼30 years) [10]. Also, they lack a sense of inflammatory pain and pain from chemical irritants including capsaicin and acid [11], [12], and they show a profound resistance to cancer [13], [14]. The present study was designed to explore yet another remarkable trait of this species: extreme brain tolerance to hypoxia [15].

Naked mole-rats are mouse-size rodents that naturally live in large colonies of up to 290 individuals in sealed subterranean burrows in northern East Africa [16]. Subterranean animals, in general, must cope with low ambient oxygen levels, due both to poor gas exchange from the surface through soil and to competition for oxygen with microorganisms and respiring plant roots [17]–[19]. Oxygen depletion (and carbon dioxide accumulation) is even more pronounced for naked mole-rats since large groups of conspecifics huddle together in nests 1.5–2.5 m underground, competing for the same poorly ventilated air [16], [19].

Consistent with this environmental challenge and a long subterranean evolutionary history dating to the Miocene [20], naked mole-rats display several physiological adaptations for survival in a chronically hypoxic environment. Notably, their hemoglobin has a higher affinity for oxygen than most other mammals [21], and their weight-specific metabolic rate is about one-third less than that of other rodents [9]. We recently reported another characteristic consistent with evolving in a hypoxic environment [15]: We found that hippocampal brain slices from adult naked mole-rats maintained synaptic transmission at low oxygen concentrations that caused transmission to decrease or cease altogether in slices from laboratory mice. Also, in nominally zero oxygen, naked mole-rat slices maintained electrophysiological function more than three times as long as slices from mice, and frequently recovered even after an anoxic depolarization lasting several minutes.

Oxygen deprivation triggers a cascade of cellular processes in neurons, including alterations in metabolic enzymes and ion channels, release of neurotransmitters such as glutamate and adenosine, and activation of receptor-coupled signaling mechanisms [22], [23]. A key element of the hypoxia cascade that determines whether the cellular response is reversible or, alternatively, leads to cell death is the accumulation of free intracellular calcium ions, which triggers cytotoxic mechanisms [24]–[26]. Therefore, the present study was undertaken to determine if the calcium response to hypoxia in hippocampal neurons is different in brain slices from naked mole-rats and mice. Mice were chosen as representatives of typical terrestrial mammals since they are not known to be especially adapted to hypoxia and have similar body size as naked mole-rats. Fura-2 was used to image intracellular calcium in slices from relatively mature (postnatal day 20, P20) naked mole-rats and mice during episodes of hypoxia.

We also made measurements of calcium accumulation in slices from early postnatal (P6) mice and naked mole-rats because age is an important factor in hypoxia tolerance. It has been known for decades that embryonic and early postnatal mammals are relatively tolerant to hypoxia. This is also of interest because other data suggest that several possibly unrelated electrophysiological processes in adult naked mole-rat brain resemble those of neonatal mice or rat brain [15]. Furthermore, factors identified as protective in neonatal rat brain generally act to limit intracellular calcium accumulation; such factors include levels of glutamate release [27]–[29], N-Methyl-D-aspartic acid (NMDA) receptor subunit composition [30], [31], Adenosine-5′-triphosphate (ATP) consumption rates, and other metabolic adaptations [26], [32], [33].

## Materials and Methods

### Animals

Experiments were performed on male and female C57BL/6 mice (bred from stock obtained from Charles River Laboratories, Wilmington, MA) and naked mole-rats of both sexes (born in colonies maintained in our laboratories) housed under normoxic laboratory conditions. Most experiments were conducted on mice and naked mole-rats at P5–7 (early postnatal) or P18–22 (late postnatal). For some experiments, we used older naked mole-rats, up to three weeks after weaning (P37–42). The total number of animals and the total number of slices for each age group, species, and experimental treatment is listed in Table 1. Note that 1 to 4 slices from each animal were used. Animal protocols were approved by the University of Illinois at Chicago Institutional Animal Care and Use Committee.

**Table 1:** Number of animals and slices used to collect data for hypoxia and high potassium.

		Mouse	Mouse	NMR	NMR	NMR	NMR
		P5–7	P18–22	P5–7	P18–22	P37–42	P5–7 (15 min)
Hypoxia	Total # animals	6	6	5	3	2	2
	Total # slices	11	11	14	10	6	7
High K^+^	Total # animals	4	5	4	4	---	---
	Total # slices	9	8	10	15	---	---

NMR = naked mole-rat.

### Slice preparation

**Figure 1:**
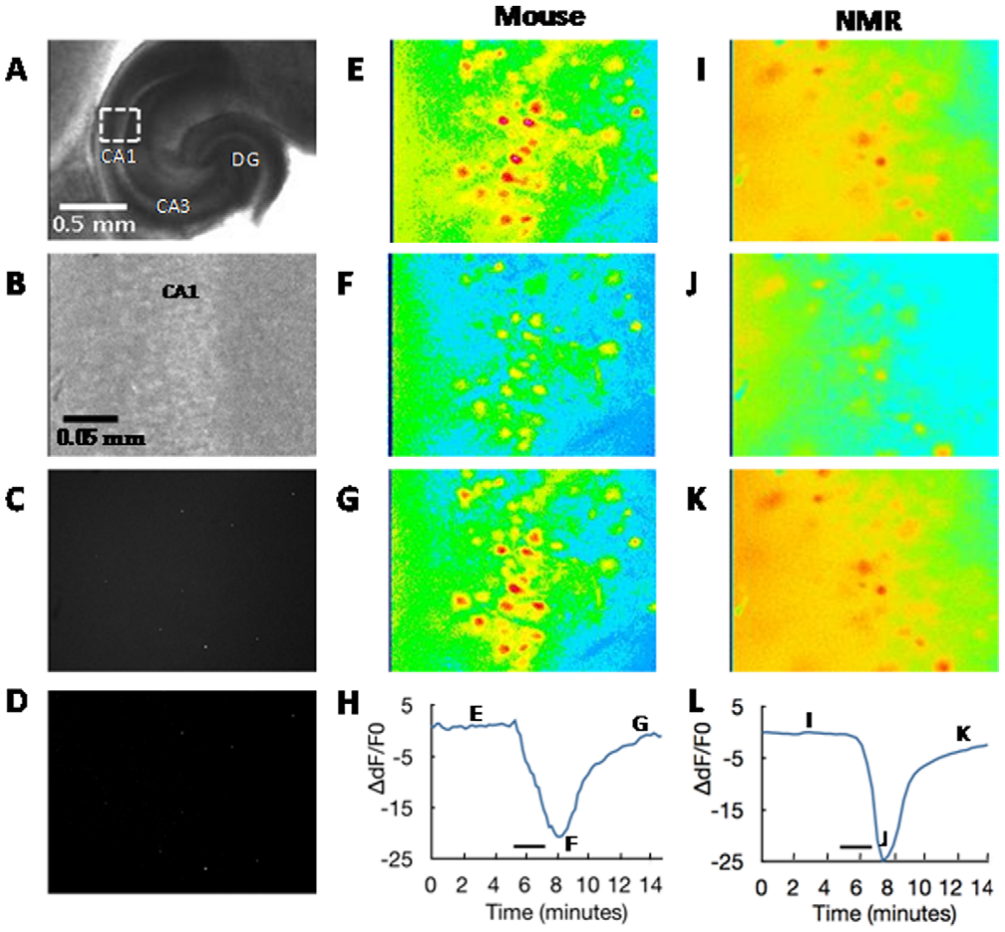
Images of hippocampal slices. **A.** Low magnification, bright field image of a slice with a box indicating the typical target area for imaging within the CA1 field of the hippocampus. DG = dentate gyrus. Top is posterior. **B.** High magnification, bright field image of a slice of the CA1 region. **C.** Same slice as shown in B, exposed to 365 nm wavelength, this slice was not loaded with fura-2 and the image reflects a 10 second integration time. **D.** Same slice as B and C, exposed to 365 nm wavelength, again not loaded with fura-2. The image reflects a 1 second integration time during exposure to hypoxia. **E–G.** Representative data from a P6 mouse slice tested with 25 mM potassium. Images show CA1 cells loaded with fura-2 before, during, and after application of 25 mM potassium for 2 minutes. The decrease in fluorescence in F corresponds to an increase in internal calcium due to application of potassium. These representative images are at 380 nm wavelength (not ratiometric). **H.** Curve showing the ratiometric data for the slice in E–G over 15 minutes. For this example, 4 minutes of data are shown prior to switching the bath solution to 25 mM potassium to illustrate the stability of typical baseline responses. For all group analyses, we collected data for 1 minute prior to switching solutions. The black bar indicates when the 25 mM solution was in the recording chamber (there was about a 1 minute time lag after switching solutions due to travel time to the recording chamber). **I–L.** An example of the same procedures on a P6 naked mole-rat.

Hippocampal brain slices from mice and naked mole-rats were prepared for fura-2 imaging as described previously [34]–[36]. Briefly, animals were anesthetized with isoflurane, decapitated, and brains were quickly removed and put into a high sucrose (70 mM) solution on ice. The chilled and submerged brain was cut on a vibratome into 300 µm slices. The slices were incubated in the high sucrose solution at 34°C for 35 minutes, then transferred to normal artificial cerebral spinal fluid (ACSF) at 34°C for 20 minutes. The ACSF contained 125 mM NaCl, 26 mM NaHCO_3_, 1.25 mM NaH_2_PO_4_, 2.5 mM KCl, 1.5 mM MgCl_2_, 2 mM CaCl_2_, 15 mM glucose, and had a pH of 7.4. Solutions were aerated with 95% O_2_/5% CO_2_. The slices were allowed to recover at room temperature for 10 minutes before staining. **Figure 1A,B** shows low and high magnification images of the Cornu Ammonis area 1 (CA1) target area.

### Fura-2 AM Staining

Slices were loaded with 5 µM membrane-permeable fura-2 AM (acetoxymethyl ester) dye (Biotium, Inc., Hayward, CA) in ACSF for 30 minutes at room temperature, while resting on a thin membrane of oxygen-permeable polydimethylsiloxane, oxygenated from below with 5% CO_2_/95% air. The slices were then incubated in ACSF at 34°C for 10 minutes and allowed to recover in ACSF at room temperature for 15 minutes. All experiments were conducted at room temperature. Except for the 30-minute loading time, ACSF solutions were aerated with 95% O_2_/5% CO_2_.

### Imaging

A stained slice was transferred into a physiological chamber mounted on an epifluorescence microscope with a 40× objective (Olympus, Center Valley, PA). The slice was submerged in ACSF bubbled with a 95% O_2_/5% CO_2_ gas mixture. The bath solution was perfused into the 1 ml chamber at a rate of 2 ml/min. The slice was positioned such that the visual field through the microscope was centered on cell bodies in the CA1 region of the hippocampus (**Figure 1B**). Measurements were made of relative internal cytosolic calcium by measuring the fura-2 fluorescence emission at 510 nm using a Cooke Sensicam CCD camera. Ratiometric data were made with 365/380 nm wavelength excitations, using a software-controlled (Imaging Workbench, Santa Clara, CA) fast wavelength changer (Sutter, Inc., Novato, CA) coupled to a metal halide source lamp (Exfo, Inc., Quebec, Canada). For group comparisons and figures, ratiometric data (380 nm intensity divided by 365 nm intensity) were converted to percent change in florescence by dividing the ratios obtained from each image by the average intensity ratio during the baseline recording period and multiplying the result by 100.

To determine if there was an appreciable background fluorescence signal, we took images of slices (n = 4) that had been prepared as described above except that they had not been loaded with the fura-2 dye. We found a negligible amount of fluorescence even with a long integration time of 10 seconds (1-second integration time was used for the experiments), and there were no structural features distinguishable (**Figure 1C**).

### Potassium Application

In one set of experiments, slices were challenged by perfusion of ACSF containing elevated concentrations of potassium. Baseline images were recorded every 20 seconds for 20 minutes prior to application of potassium to ensure that the slice was healthy and that movement was negligible. For potassium application, the bath solution was switched to one with a high concentration of potassium (equimolar replacement of NaCl with KCl). In pilot tests with potassium concentrations ranging from 5 to 30 mM, we found that 15 and 25 mM were well on the dynamic part of the concentration/response curve for slices from P6 mice (which had the maximum response) and did not saturate the fura-2 indicator response. Based on that, we collected a complete data set for both 15 and 25 mM potassium. Images were collected every 20 seconds for 15 minutes beginning 1 or 4 minutes before switching to one of the high potassium solutions. After 2 minutes, the solution was switched back to normal bath solution and the slice was allowed to recover. Ten minutes after recovery, the other high potassium solution was applied for 2 minutes followed by recovery. The order of potassium solutions was alternated between slices. Pseudocolor example images of fluorescence at 380 nm taken before, during, and after application of 25 mM potassium are shown in **Figure 1E, F, G** for a P6 mouse and **I, J, K** for a P6 naked mole-rat. The curves showing ratiometric data for all 15 minutes of testing are shown in **Figure 1H and L**. Note that in these particular example slices, we collected data for 4 minutes prior to switching to a high potassium solution. This duration of baseline illustrates the stability of the response. Data collected for group analyses used a baseline duration of 1 minute.

### Hypoxia

In another set of experiments, slices were challenged with ACSF depleted of oxygen (hypoxia). After collecting baseline images, hypoxia was induced by switching the bath solution from the one saturated with 95% O_2_/5% CO_2_ to one saturated with 95% N_2_/5% CO_2_ for 10 minutes.

Ten minutes of hypoxia was chosen based on pilot experiments. For durations of hypoxia lasting longer than 10 minutes, most mouse slices did not show an appreciable recovery, and our aim was to induce a reversible effect. A second issue with longer periods of hypoxia was that the fluorescent signal saturated. Our aim was to avoid saturation because there is no way to distinguish saturation of calcium concentration versus saturation of the fluorescent signal (the fluorescent signal can saturate before the calcium concentration saturates).

A series of images was recorded every 20 seconds for 20 minutes beginning 1 minute before switching to the hypoxic solution. After 10 minutes, the bath solution was switched back to the one saturated with 95% O_2_.

To determine if autofluorescence increased during hypoxia exposure, for instance from increased NADH production, we subjected unstained slices (n = 4) to the hypoxia exposure protocol. We found a negligible amount of fluorescence, and there were no structural features distinguishable (**Figure 1D**).

### Statistical Analysis

We used a 2-way ANOVA (GB STAT, Dynamic Microsystems, Inc., Silver Spring, MD) for comparisons involving more than two groups (e.g. species and age), followed by the Newman-Keuls test for multiple comparisons. The values used for statistical analyses were from the last data point collected under exposure to hypoxia or high potassium.

In addition, we used a two-tailed, unpaired t-test (Excel) to compare the late postnatal (P18–22) naked mole-rat slices with slices from a group of even older naked mole-rats (P37–42).

## Results

### Hypoxia-induced increase in internal calcium is reduced in naked mole-rat compared to mouse

As predicted, hippocampal CA1 pyramidal cells of both naked mole-rats and mice responded to hypoxia with a decrease in fura-2 fluorescence, corresponding to an increase in intracellular calcium. However, there were statistically significant differences in the fluorescence response due to main effects of species (**F**_1,45_ = 52.61, *p*<.0001) and age (**F**_1,45_ = 18.96, *p*<0.0001) (2-way ANOVA). There was no significant interaction between age and species (**F**_1,45_ = 0.37, *p* = .55).

**Figure 2:**
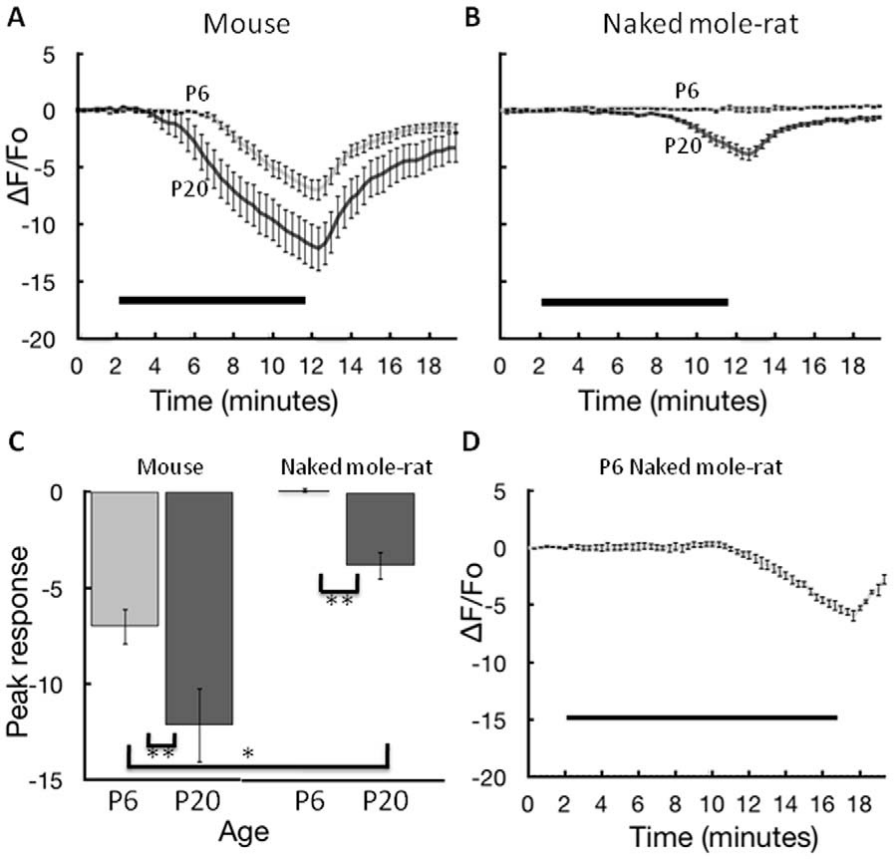
Increase in internal calcium from exposure to hypoxic bath solution. **A.** Data from P6 (11 slices, 6 animals) and P20 (11 slices, 6 animals) mouse hippocampal slices. Values on the y-axis indicate the percent change in calcium-mediated fluorescence within CA1 neurons in the field of interest with negative values corresponding to an increase in calcium (calcium decreases the fluorescent signal). Images were collected every 20 seconds over 20 minutes. The black bar indicates the 10 minutes when hypoxic bath solution was in the recording chamber. Error bars are +/− S.E.M. **B.** Data from P6 (14 slices, 5 animals) and P20 (10 slices, 3 animals) naked mole-rat slices. **C.** Summary data showing the change in maximal calcium with age for mice and naked mole-rats for a 10-minute exposure. * and ** correspond to significance at p<0.05 and p<.01, respectively according to the Newman-Keuls test. **D.** Data from P6 (7 slices, 2 animals) naked mole-rats slices with an extended hypoxia exposure (15 minutes). Note that in all panels, animals in the P6 groups actually ranged in age from P5 to P7, and animals in the P20 groups actually ranged in age from P18 to P22.

The data from mouse slices were consistent with previous studies of mouse, rat, and gerbil [37]–[41]. Mouse slices showed a progressive increase in internal calcium (decrease in fluorescence signal) beginning shortly after application of hypoxic bath solution and continuing for the entire 10 minutes of hypoxia exposure (**Figure 2A**). Calcium then decreased after re-oxygenation. As expected, slices from neonatal mice showed a weaker increase than slices from older mice. The maximal percent change in fluorescence signal for slices from older mice (P18–22) was −12.1% (mean)+/−1.96% (SE), whereas the maximal percent change for slices from younger mice (P5–7) was −6.9+/−0.93% (*p*<.01, Newman-Keuls test). Note that both of these values could have probably reached higher values if we had used a longer exposure to hypoxia, but that would have precluded an appreciable recovery (see materials and methods). Therefore, it is the differences between groups that are important, not the absolute values.

Naked mole-rat slices tested with the same procedure showed a much smaller change in intracellular calcium-mediated fluorescence (**Figure 2B**). The slices from older naked mole-rats had a maximal percent change of −3.8+/−0.75%, which was significantly less than both the older (*p*<0.01, Newman-Keuls test) and younger (*p*<0.05, Newman-Keuls test) mouse groups. Remarkably, the slices from neonatal naked mole-rats showed no detectable change in internal calcium during the entire 10-minute hypoxia exposure. The summary data for both age groups of naked mole-rats and mice are presented in **Figure 2C**.

Historically, the age groups we tested are considered to be representative of neonatal (P5–7) and mature (P18–22) animals in regard to responses to hypoxia in hippocampal slices [31], [38]. We tested both mice and naked mole-rats at the identical chronological ages. However, it should be noted that the maximum lifespan in naked mole-rats (30 years) is much longer than that in mice (3 years), raising the possibility that chronological and biological age diverge in the two species. On the other hand, both naked mole-rats and mice reach a major developmental milestone (weaning) at about the same age (3–4 weeks), suggesting a comparable level of overall maturity at this time point. In any case, we addressed the issue of biological maturity by testing a group of naked mole-rats at P37–42, well after weaning. The responses to hypoxia for these naked mole-rats were not significantly different from the P20 group (maximal percent change for P37–42 = −3.82+/−0.49%, maximal percent change for P18–22 = −3.80+/−0.75%, *p* = 0.959, t-test). These results suggest that the difference in hypoxia tolerance between mice and naked mole-rats at P20 is not simply due to a difference in rate of maturation. Both mice and naked mole-rats show hypoxia tolerance in the neonatal period (P5–7), but hypoxia sensitivity reaches adult levels by P18–22. The calcium response to hypoxia is significantly reduced in naked mole-rat neurons relative to mouse neurons both early and late in development.

It is also notable that the uptake of the fura-2 AM dye was not qualitatively different in slices from P37–42 and P18–22 naked mole rats, whereas staining of mice older than P22 was poor and inconsistent.

The dramatic results for slices from neonatal (P5–7) naked mole-rats warranted further testing on this age group. In our experiments, the 10-minute duration of hypoxia was selected based on pilot studies with mouse slices and was somewhat arbitrary. Therefore, we tested slices from neonatal naked mole-rats with a longer exposure to hypoxia (15 minutes). During this longer period of hypoxia, slices from naked mole-rats began to show an increase in calcium, but not until much later than the other groups (**Figure 2D**).

### Potassium-induced increase in internal calcium is similar in older groups of naked mole-rats and mice

**Figure 3:**
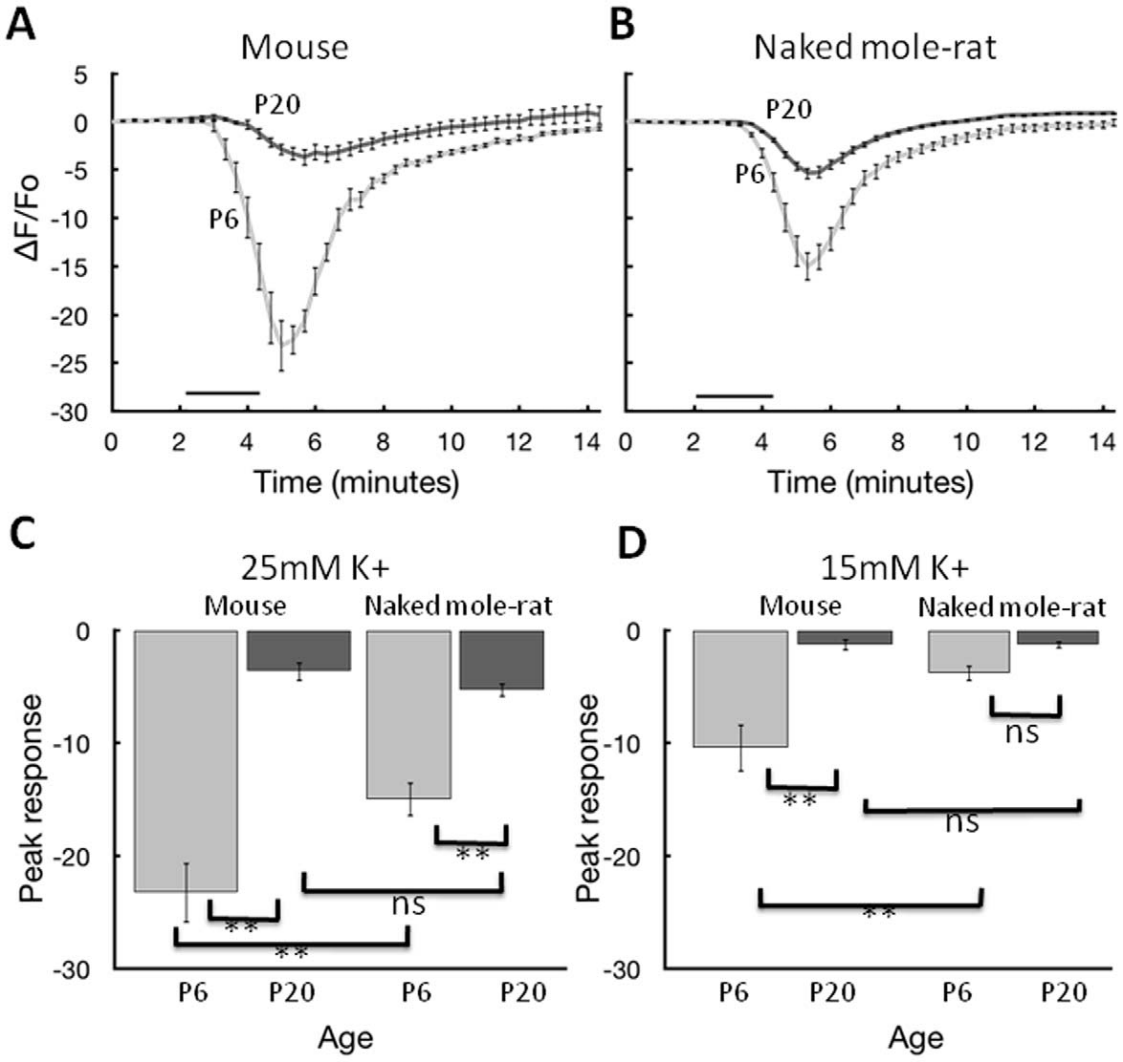
Increase in internal calcium from exposure to high K+ bath solutions. **A.** Data from P6 (9 slices, 4 animals) and P20 (8 slices, 5 animals) mouse hippocampal slices. Images were collected over 15 minutes. The black bars indicate the 2 minute time course that 25 mM potassium bath solution was applied. **B.** Data from P6 (10 slices, 4 animals) and P20 (15 slices, 4 animals) naked mole-rat slices tested under the same conditions as A. **C.** Summary data showing the change in maximal calcium with age for P6 and P20 mice and naked mole-rats during the 2 minute exposure to 25 mM K+. **D.** Summary data showing the change in maximal calcium with age for P6 and P20 mice and naked mole-rats during a 2 minute exposure to 15 mM K+. * and ** correspond to significance at p<0.05 and p<.01, respectively according to the Newman-Keuls test.

The blunted calcium response to hypoxia in naked mole-rat slices could be due to a number of factors (calcium channel density, buffering capacity, etc.), not related to hypoxia *per se*. To explore this issue further, calcium signals in naked mole-rat and mouse slices were measured during challenges with elevated extracellular potassium concentrations. **Figure 3A–C** presents the data for experiments in which slices from mice and naked mole-rats (both at P6 and P20) were exposed to 25 mM potassium. A 2-minute exposure to 25 mM potassium triggered an increase in intracellular calcium in both mice and naked mole-rats (**Figure 3A,B**). In both species, the response was much greater in the younger age group: 6 times greater in mice and 3 times greater in naked mole-rats (**Figure 3C**). For mice, the maximal percent change in fluorescence signal for slices from older mice (P18–22) was −3.64+/−0.88%, whereas the maximal percent change for slices from younger mice (P5–7) was −23.19+/−2.6% (*p*<.01, Newman-Keuls test). For naked mole-rats, the maximal percent change in fluorescence signal for slices from older naked mole-rats (P18–22) was −5.29+/−0.65%, whereas the maximal percent change for slices from younger naked mole-rats (P5–7) was −15.0+/−1.51% (*p*<.01, Newman-Keuls test). Using a 2-way ANOVA, we found significant differences between age groups (**F**_1,38_ = 93.69 *p*<0.0001), and between species (**F**_1,38_ = 4.72, *p*<.05), and a significant interaction between age and species (**F**_1,38_ = 10.49, *p*<0.01). With a lower dose of potassium (15 mM), all responses were reduced but the same patterns among age groups and species remained (**Figure 3D**): Significant differences were found between age groups (**F**_1,38_) = 30.67,*p*<0.0001), and between species (**F**_1,38_ = 10.17, *p*<.05), as well as a significant interaction between age and species (**F**_1,38_ = 10.22, *p*<0.01).

There are several noteworthy aspects of these data. The potassium challenge demonstrated a concentration-dependent increase in fluorescence that was greater in some cases than the responses due to hypoxia, suggesting that the hypoxia measurements were well within the dynamic range of the fura-2 indicator. In addition, the responses to potassium challenge were much more similar for naked mole-rats and mice than the responses to hypoxia; this was particularly evident for the older age groups where the potassium responses were not significantly different. This is important because it shows that the blunted calcium response to hypoxia in naked mole-rats cannot be entirely accounted for by a generalized reduction in responsiveness to all stimuli. Furthermore, it suggests that the differences we found were not due to species differences in dye uptake which in our experience is most problematic in older mice.

Finally, the older age groups had a smaller calcium accumulation response to potassium challenge than the younger age groups in both mouse and naked mole-rat, which is the opposite pattern from the one we observed with hypoxia. This is important because it suggests that the age differences we found for hypoxia were not due to poor slice health in older animals.

## Discussion

The main finding of this study is that hippocampal neurons in naked mole-rats show a blunted intracellular calcium response to hypoxia when compared to neurons in mice. The attenuated calcium accumulation response in naked mole-rat hippocampus was highly significant compared to the response in mouse hippocampus, whether assessed in animals at an early stage of postnatal development (P6) or in weanlings (P20). Technical issues did not permit a comparison of calcium responses in adult mice and naked mole-rats because fura-2 AM staining of adult tissue is impaired as the neuropil develops; however a previous electrophysiological study demonstrated that adult (>1 year old) naked mole-rat hippocampus is extremely tolerant to two related effects of hypoxia: namely, the suppression of synaptic transmission during partial hypoxia and the collapse of membrane potentials (anoxic depolarization) that accompanies severe oxygen deprivation [15].

We chose to examine hypoxia responses in acute brain slices rather than cell culture because the slice represents more closely the neuronal circuits and neural-glial ensembles present *in situ* and we wanted to compare responses in tissue from animals at different ages. However, we are limited to inferring relative changes in calcium, in this case in an ensemble of neurons in our region of interest, in slices bulk-loaded with the calcium indicator. Absolute calibration of indicator-based calcium measurements is technically difficult under optimal conditions (isolated cells, individually loaded), and even then interpretation can be problematic [42]. It was therefore not possible to calibrate absolute calcium changes accurately in our bulk-loaded slices.

The attenuation of calcium response in naked mole-rat hippocampus appears to be specifically related to hypoxia tolerance because the calcium response to potassium challenge was much more similar for naked mole-rats and mice. First, both mice and naked mole-rat neurons showed an age-dependent *reduction* in calcium response to potassium rather than the age-dependent *increase* in calcium response to hypoxia. Second, at P20, naked mole-rat neurons showed a highly significant reduction (68%) in calcium response to hypoxia compared to mouse neurons, but no significant difference in calcium response to potassium compared to mouse neurons.

A diminished accumulation of intracellular calcium during hypoxia is a common end point in hypoxia tolerance under a variety of conditions [26], [43]. For example, during hibernation, Arctic ground squirrels have less phosphorylation of the NR1 subunit of NMDA receptors; this effect decreases the activity of the receptor, thereby limiting calcium accumulation [44], [45]. Similarly, western painted turtles survive long periods of anoxia (months) using a variety of mechanisms, including down-regulation of ion channels and suppression of glutamate release. These adaptations limit calcium accumulation in neurons (e.g., [46], [47]). In hypoxia-tolerant neonatal rats, hippocampal neurons have elevated levels of the NMDA receptor subunit, NR2D, compared to adult rats; this subunit is less sensitive to hypoxia than the other NMDA receptor subunits [26], [31]. Metabolic differences [33] may also indirectly tend to limit calcium accumulation during hypoxia in neonates compared to adults.

Naked mole-rats share two important features with these other model systems: resistance to hypoxia *in vivo* and an attenuated hypoxia-induced neuronal calcium response *in vitro*. We do not yet know the underlying mechanism(s) behind the extreme tolerance to hypoxia in naked mole-rat neurons. However, it appears that, unlike the turtle [48], an increase in adenosine does not contribute to the diminished increase in calcium. In a previous study, we showed that hippocampal cells in adult naked mole-rats were less sensitive to adenosine as compared to cells from mice [15]. We suggested that the adult naked mole-rat brain resembles the neonatal rat (and mouse) brain in terms of response to adenosine, resistance to hypoxia, and lack of paired-pulse facilitation [49]. (The robust staining of naked mole-rat neurons with fura-2 AM at P42, an age when staining of mouse or rat neurons is poor and inconsistent, may also reflect a difference in neuronal maturation.) Furthermore, we recently showed that adult naked mole-rat brain retains more of the (neonatally abundant) NMDA receptor subunit, NR2D, compared to mice [50]. The calcium imaging results from the present study are not inconsistent with the notion that slowed or arrested brain development may endow the naked mole-rat brain with extreme hypoxia tolerance. Even at P42 (the most advanced age tested), naked mole-rat brain showed an attenuated calcium accumulation response to hypoxia, compared to neonatal (P6) mice.

Even though naked mole-rats showed a blunted response to hypoxia compared to mice at all ages, the naked mole-rats also showed a significant age-related increase in the response from P6 to P20. It is possible that naked mole-rats undergo a much attenuated change with age (compared to mice), retaining a substantial, although incomplete, degree of “neonatal” hypoxia tolerance into adulthood. Alternatively, the naked mole-rat and mouse may have a comparable age-related *change* in hypoxia sensitivity, but the naked mole-rats have such an extremely diminished response to begin with (at P6) that the change is not large enough to bring older animals into the response range of even the P6 mice. In any case, the calcium accumulation response to hypoxia was significantly reduced in naked mole-rats at P20 even compared to P6 mice, and did not show further changes from P20 to P40.

Our working hypothesis is that naked mole-rats have evolved an extreme tolerance to hypoxia as a consequence of their unusual lifestyle, which combines subterranean living with a proclivity for living in great numbers (up to hundreds of individuals per colony). Hence, even compared to other fossorial mammals, naked mole-rats are challenged by unusually high levels of hypoxia due to many individuals sharing the same poorly ventilated air [19]. The current thought on what has driven the naked mole-rat to live the way it does is based on its habitat - hard soil which makes burrowing costly, and patchy food resources (roots and tubers), which make foraging by a small number of animals risky (food-aridity hypothesis [51]). Consistent with the notion that naked mole-rats are hypoxia-tolerant even among fossorial mammals, a previous study found that hippocampal slices from another fossorial mammal, the blind mole-rat (*Spalax*), which primarily lives a solitary life, responded to severe hypoxia much more similarly to slices from mice than slices from naked mole-rat. Under severe hypoxia, hippocampal slices from naked mole-rats maintained synaptic function for 12.63+/−5.06 minutes, whereas slices from mice maintained function for 2.16+/−0.23 minutes and slices from blind mole-rats maintained function for 1.58+/−0.17 minutes [15]. This was an interesting finding because the blind mole-rat is considered to be a hypoxia tolerant animal *in vivo*, with a variety of physiological and anatomical adaptations in blood and respiratory organs, and a number of gene products which are consistent with living under hypoxic conditions (see [52] for review). Apparently, this fossorial species achieves hypoxia tolerance via a variety of mechanism that do not include intrinsic brain tolerance.

Currently there are relatively few comparative studies on intrinsic hypoxia tolerance in brain slices in mammals. However, one such study measured membrane potentials from visual cortex slices from diving seals and mice [53]. They found that under severe hypoxia, slices from seals maintained synaptic function approximately 4 times longer than slices from mice (19 minutes versus 5 minutes). This is similar to what we found previously when comparing hippocampal slices from naked mole-rats and mice (12.63 minutes versus 2.16 minutes [15]). Using cell survival as a metric, another study [54] showed that CA1 cells in hippocampal slices from both hibernating and active 13-lined ground squirrels survived longer than CA1 cells in slices from rats. Consistent with this finding, a variety of hypoxia tolerant adaptations have been found in brain cells of hibernating species (see [43] for review). In our present study, we did not look at cell survival because our protocol was designed to measure recovery. However, our previous study which measured physiological responses to hypoxia clearly showed that hippocampal slices from naked mole-rats were able to survive and/or recover from hypoxia applications that slices from mice could not recover from [15]. Non-mammalian models of hypoxia tolerance include some fishes, frogs, and turtles [55]. Interestingly, forebrain cells from tadpoles [56] and cortical slices from turtles [55] show increases in internal calcium during severe but survivable hypoxia without showing the cell damage characteristic of mammalian neurons exposed to high calcium.

In the present study, the animals that we used were maintained under normoxic conditions prior to slicing. It is intriguing to speculate about the possible effects of maintaining the animals under moderately hypoxic conditions that might simulate conditions within a naked mole-rat burrow. Periods of moderate hypoxia are well known to increase hypoxia tolerance in a variety of species ([57] for review). Future experiments using this type of preconditioning may reveal an even more pronounced blunting of the neuronal calcium response to hypoxia in naked mole-rat neurons.

In summary, hippocampal neurons from naked mole-rats show an attenuated intracellular calcium accumulation response to hypoxia, as compared with mouse hippocampal neurons. The blunted calcium response may represent an important adaptive response to a chronic hypoxic environment that is achieved by retarding or arresting a developmental process that normally limits hypoxia tolerance in adult mammals.
